# Tamoxifen-induced knockdown of the mitochondrial calcium uniporter in Thy1-expressing neurons protects mice from hypoxic/ischemic brain injury

**DOI:** 10.1038/s41419-018-0607-9

**Published:** 2018-05-22

**Authors:** Matthew Nichols, Evgeny V. Pavlov, George S. Robertson

**Affiliations:** 1 Department of Pharmacology, Brain Repair Centre, Faculty of Medicine, 2nd Floor, Life Sciences Research Institute, Dalhousie University, 1348 Summer Street, P.O. Box 15000, Halifax, Nova Scotia Canada B3H 4R2; 2 Department of Basic Sciences, College of Dentistry, New York University, 345 East 24th Street, New York, NY 10010 USA; 3 Department of Psychiatry, QEII Health Sciences Centre, 5909 Veterans’ Memorial Lane, 8th floor, Abbie J. Lane Memorial Building, Halifax, NS B3H 2E2 Canada

## Abstract

The mitochondrial calcium uniporter (MCU) mediates high-capacity mitochondrial calcium uptake that stimulates energy production. However, excessive MCU activity can cause ischemic heart injury. To examine if the MCU is also involved in hypoxic/ischemic (HI) brain injury, we have generated conditional MCU knockout mice by tamoxifen (TMX) administration to adult MCU-floxed (MCU^fl/fl^) mice expressing a construct encoding Thy1-cre/ERT2-eYFP. Relative to TMX/Thy1-cre/ERT2-eYFP controls, HI-induced sensorimotor deficits, forebrain neuron loss and mitochondrial damage were decreased for conditional MCU knockout mice. MCU knockdown by siRNA-induced silencing in cortical neuron cultures also reduced cell death and mitochondrial respiratory deficits following oxygen-glucose deprivation. Furthermore, MCU silencing did not produce metabolic abnormalities in cortical neurons observed previously for global MCU nulls that increased reliance on glycolysis for energy production. Based on these findings, we propose that brain-penetrant MCU inhibitors have strong potential to be well-tolerated and highly-efficacious neuroprotectants for the acute management of ischemic stroke.

## Introduction

Neurons depend heavily on mitochondria to buffer cytosolic calcium (Ca^2+^) concentrations and meet the dynamic metabolic demands imposed by neurotransmission^1–3^. However, mitochondria can also trigger neuronal cell death. Excessive mitochondrial Ca^2+^ uptake initiates the formation of a mitochondrial membrane permeability transition pore (mPTP) that executes both apoptotic^4,5^ and necrotic^6–9^ neuronal cell death. Identification of the mitochondrial Ca^2+^ transport mechanisms that trigger ischemic neuronal cell death may thus open new therapeutic avenues for mitigating brain damage associated with ischemic stroke^10–12^.

The mitochondrial Ca^2+^ uniporter (MCU) is responsible for rapid and high-capacity mitochondrial Ca^2+^ uptake in the heart^13^. Genetic identification of the MCU in 2011^14,15^ has enabled the generation of various genetic mouse lines in which MCU activity is blocked by either global MCU (G-MCU) deletion^13^ or cardiac-specific expression of a dominant-negative MCU (DN-MCU)^16,17^ or inducible cardiac-specific MCU ablation at maturity^18,19^. Experimentation with these genetic lines has shown that conditional, but not constitutive (G-MCU nulls or DN-MCU mice), MCU inhibition protects the heart from ischemic/reperfusion injury^13,16–19^. However, the precise nature of the compensations that comprise the resistance of G-MCU nulls to ischemic injury are unclear.

Given the considerable implications of these findings for ischemic neuronal cell death, we recently examined the effects of G-MCU deletion on hypoxic/ischemic (HI) brain injury^20^. Consistent with the failure of constitutive MCU inhibition to reduce ischemic heart damage, G-MCU nulls were not protected from sensorimotor deficits or neuronal damage following HI brain injury^20^. Relative to wild-type (WT) cortical neurons, energetic stress enhanced glycolysis in G-MCU null neurons that was accompanied by depressed Complex I activity. HI reduced forebrain nicotinamide adenine dinucleotide (NADH) levels more in G-MCU nulls than WT mice, suggesting that increased glycolytic consumption of NADH suppressed Complex I activity. The resultant energetic collapse may thus promote ischemic/reperfusion injury despite reduced mitochondrial Ca^2+^ uptake^20^. To avoid these compensations, we have generated a novel transgenic line enabling the MCU to be selectively deleted at maturity in forebrain neurons. We show that conditional MCU deletion in Thy1-expressing neurons renders mice resistant to HI brain injury without producing metabolic compensations observed in G-MCU nulls.

## Results

### Conditional MCU knockout in Thy1-expressing neurons attenuates HI-induced sensorimotor deficits and brain damage

**Fig. 1:**
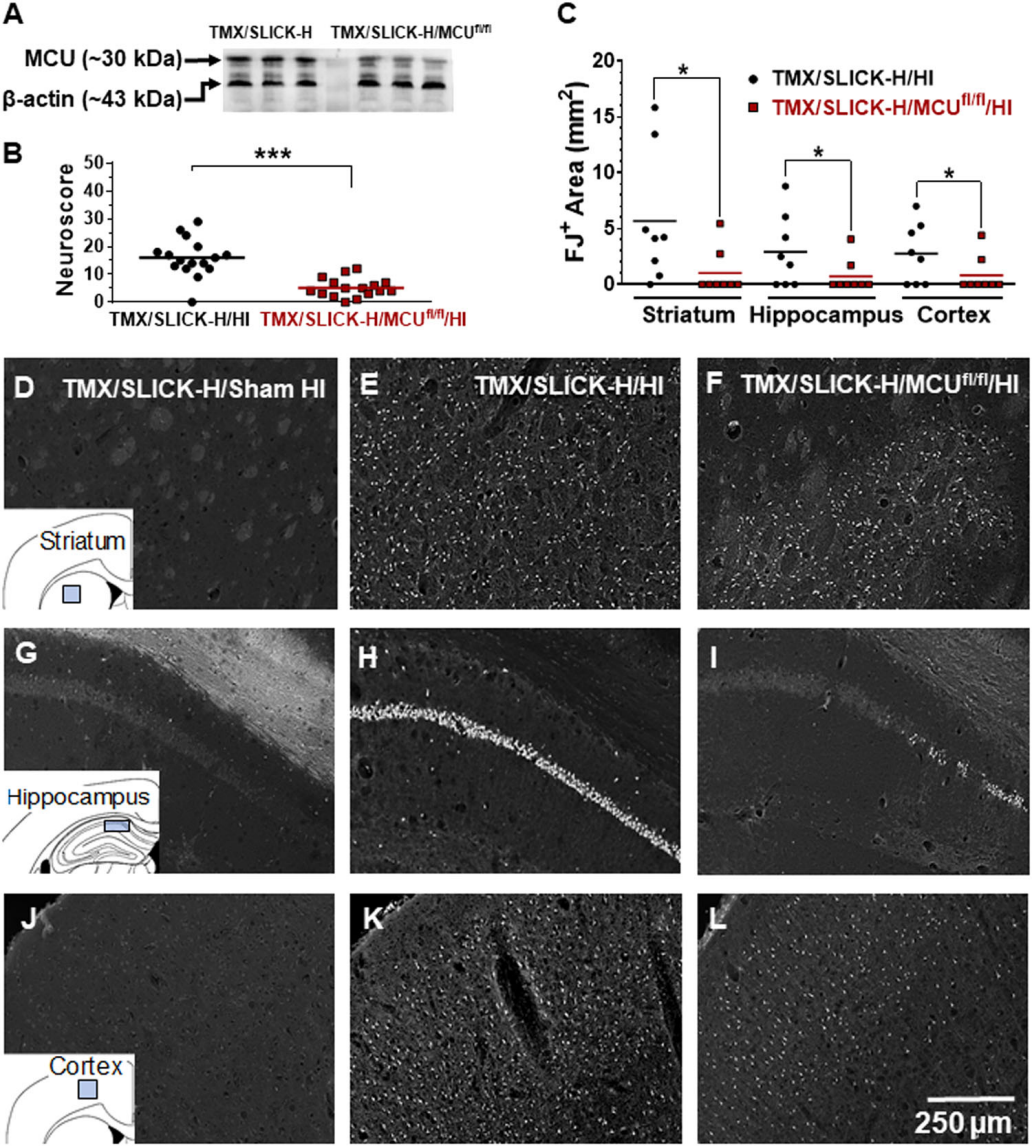
Tamoxifen-treated SLICK-H/MCU^fl/fl^ (TMX/SLICK-H/MCU^fl/fl^) mice are protected from HI-induced motor deficits and neuronal damage. **a** MCU protein levels for TMX/SLICK-H and TMX/SLICK-H/MCU^fl/fl^ mice. **b** Neuroscores for TMX/SLICK-H and TMX/SLICK-H/MCU^fl/fl^ mice after HI brain injury (TMX/SLICK-H/HI and TMX/SLICK-H/MCU^fl/fl^/HI). Mice were graded on a 56-point scale that rated increased levels of neurobehavioural impairment. **c** Quantification of Fluoro-Jade (FJ)-positive neurons damaged by HI brain injury in the dorsolateral striatum, CA1 region of the dorsal hippocampus, and anterior motor cortex of TMX/SLICK-H/HI and TMX/SLICK-H/MCU^fl/fl^/HI mice. **d**–**l** Representative images of FJ-positive neurons in a TMX/SLICK-H mouse 24 h after sham HI surgery (TMX/SLICK-H/Sham HI; **d**, **g**, and **j**) or TMX/SLICK-H and TMX/SLICK-H/MCU^fl/fl^ mice 24 h after HI (TMX/SLICK-H/HI; **e**, **h**, and **k**; TMX/SLICK-H/MCU^fl/fl^/HI; **f**, **i**, and **l**) in the dorsolateral striatum, CA1 region of the dorsal hippocampus and motor cortex. HI damage was quantified by determining the area occupied by FJ-positive neurons within the indicated sectors of dorsolateral striatum, dorsal hippocampus and anterior motor cortex (blue box for each of the inserts for **d**, **g**, and **j**). *p < 0.05, **p < 0.01, Mann–Whitney *U*-tests

SLICK-H transgenics expressing a Thy1-cre/ERT2-eYFP construct^21^ were crossed with C57Bl/6 MCU-floxed (MCU^fl/fl^) mice^18^ to generate Thy1-cre/ERT2-eYFP^+/-^/MCU^fl/fl^ (SLICK-H/MCU^fl/fl^) animals. MCU deletion in SLICK-H/MCU^fl/fl^ mice was induced at 10 weeks of age by the oral administration of tamoxifen (TMX; 80 mg/kg; once daily for 5 days). Western blotting performed 3 weeks later showed that relative to TMX-treated SLICK-H (TMX/SLICK-H) mice, MCU levels in the forebrain were reduced by ~ 50% in TMX/SLICK-H/MCU^fl/fl^ mice (Fig. 1a). This degree of neuronal MCU suppression was sufficient to reduce sensorimotor deficits 24 h following HI relative to TMX/SLICK-H/HI mice. Figure 1b shows the neuroscores for TMX/SLICK-H/HI and TMX/SLICK-H/MCU^fl/fl^ mice (*n* = 16/group) subsequently processed for FJ staining (Fig. 1d–k; *n* = 8/group) or triphenyltetrazolium chloride (TTC) staining (*n* = 8/group; Fig. 3a). Preserved sensorimotor function for TMX/SLICK-H/MCU^fl/fl^/HI mice was accompanied by decreased neuronal damage detected by FJ staining in the CA1 region of the dorsal hippocampus, dorsolateral striatum, and anterior motor cortex compared with controls animals (Fig. 1c–k).

### HI-induced neuronal cell loss in layer V was prevented in TMX/SLICK-H/MCU^fl/fl^ mice

**Fig. 2:**
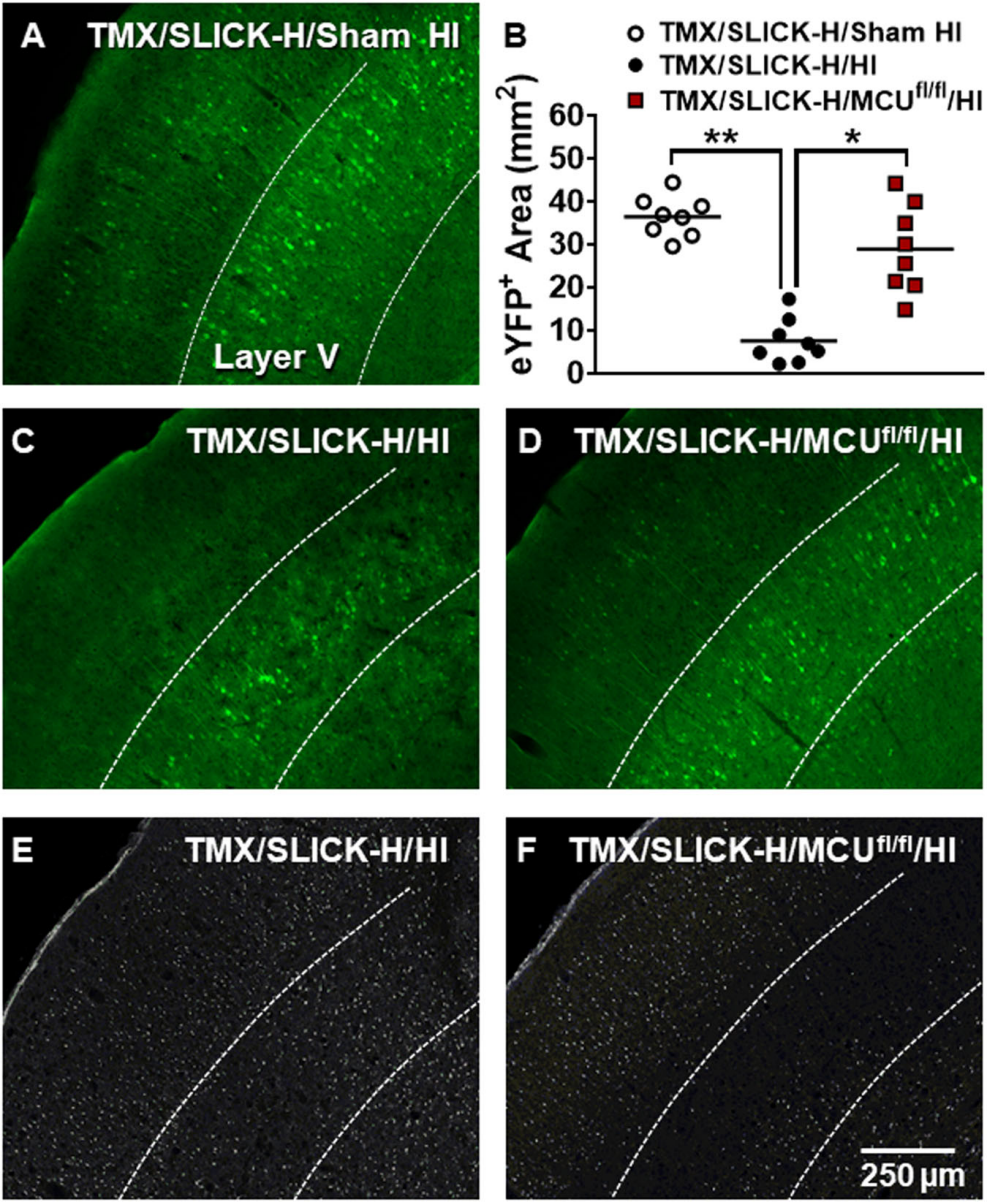
Neuronal MCU deficiency reduces the loss of eYFP^+^ neurons in the anterior motor cortex. Prominent eYFP^+^ labeling was detected in layer V of the anterior motor cortex with fewer eYFP^+^ neurons observed in layers II–IV and V **a**. The areas occupied by eYFP^+^ neurons were quantified in the region indicated by the blue box for the insert in Fig. 1J for TMX/SLICK-H and TMX/SLICK-H/MCU^fl/fl^ mice 24 h after sham HI or HI brain injury **b**. Relative to the representative TMX/SLICK-H/HI mouse **c**, eYFP^+^ neurons were preserved in the representative SLICK-H/MCU^fl/fl^/HI mouse **d**. Adjacent sections show that the reductions in FJ^+^ cells were greatest in layer V of the anterior motor cortex of the TMX/SLICK-H/MCU^fl/fl^/HI mouse **e** and **f**

We also quantified eYFP positive (eYFP^+^) neurons in TMX/SLICK-H controls and TMX/SLICK-H/MCU^fl/fl^ mice in layer V of the motor cortex after sham HI or HI brain injury (Fig. 2b). This was done by measuring the areas occupied by eYFP^+^ neurons within the anterior motor cortex (Fig. 1j, blue box of insert). Relative to TMX/SLICK-H/sham HI mice (Fig. 2a), eYFP^+^ staining was reduced by ~ 80% in SLICK-H/HI mice (Fig. 2b, c). This loss of eYFP^+^ neurons in layer V pyramidal neurons was markedly reduced in TMX/SLICK-H/MCU^fl/fl^/HI mice (Fig. 2d). Fluoro-Jade positive (FJ^+^) cells are visible in layer V of an adjacent brain section of the representative control-HI mouse (Fig. 2e). By comparison, there was a marked reduction of FJ^+^ cells in layer V of the representative TMX/SLICK-H/MCU^fl/fl^/HI mouse (Fig. 2f).

### Reduced infarct volume for TMX/SLICK-H/MCU^fl/fl^/HI relative to TMX/SLICK-H/HI mice

**Fig. 3:**
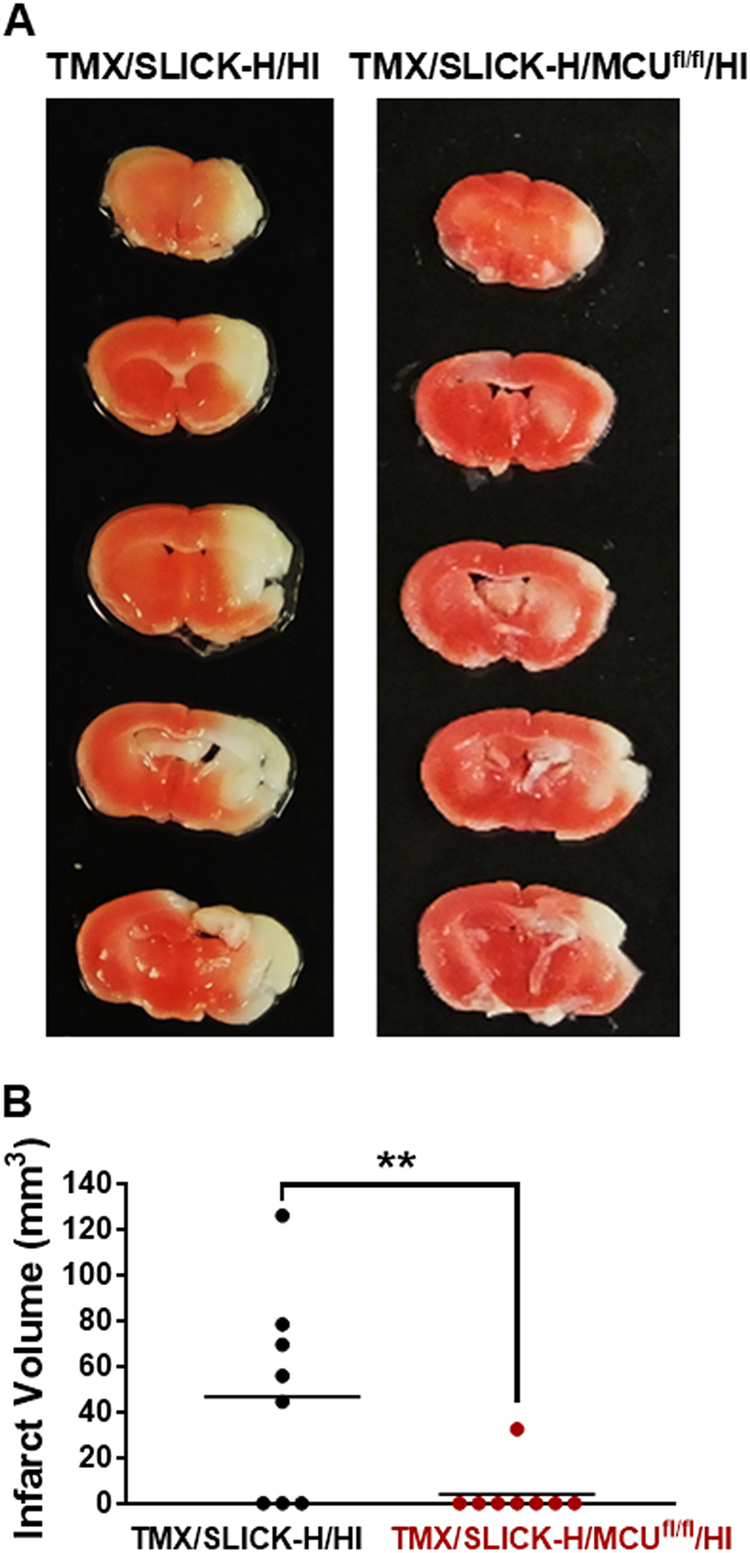
Infarct volume is reduced in TMX/SLICK-H/MCU^fl/fl^/HI mice relative to TMX/SLICK-H/HI mice. TTC staining 24 h after HI for a representative TMX/SLICK-H/MCU^fl/fl^/HI and SLICK-H/HI mice showing that neuronal MCU deficiency reduced HI brain injury **a**. Measurement of infarct volume for TTC staining demonstrated that TMX/SLICK-H/MCU^fl/fl^/HI mice sustained smaller infarcts than TMX/SLICK-H/HI mice **b**. **p < 0.01, Mann–Whitney *U*-tests

To further assess the protective effects of MCU knockout in Thy1-expressing neurons against HI brain injury, infarct volume was measured by TTC staining for TMX/SLICK-H/MCU^fl/fl^ and TMX/SLICK-H mice 24 h after HI. Relative to TMX/SLICK-H/HI mice, TMX/SLICK-H/MCU^fl/fl^/HI mice showed a smaller infarct volume further supporting the neuroprotective effects of reduced neuronal mitochondrial Ca^2+^ uptake against HI brain injury (Fig. 3a, b).

### Conditional MCU knockout in Thy1-expressing neurons protects neuronal mitochondria from HI-induced damage

**Fig. 4:**
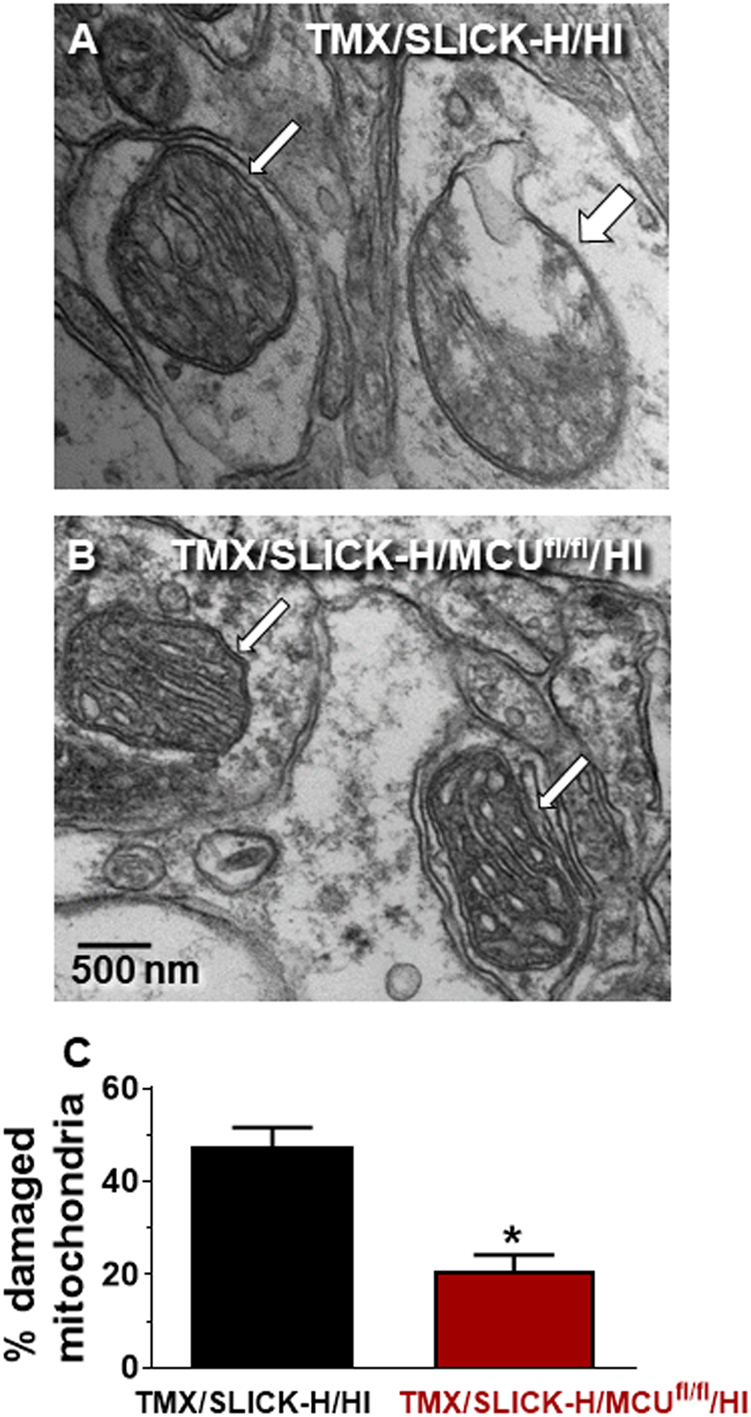
Representative EM images showing intact (thin arrows) or damaged (thick arrow) mitochondria. EM images of mitochondria within neurons of the CA1 region of the dorsal hippocampus 24 h after HI in a TMX/SLICK-H/HI (**a**) and TMX/SLICK-H/MCU^fl/fl^/HI mouse **b**. Quantification of mitochondrial damage in the CA1 region of the dorsal hippocampus 24 h after HI in TMX/SLICK-H/HI and TMX/SLICK-H/MCU^fl/fl^/HI mice, *n* = 5 (C). *p < 0.05 relative to TMX/SLICK-H/HI mice, Mann–Whitney *U*-test

Ten images collected at random (× 30,000 mag) were obtained from 5 mice in both the TMX/SLICK-H and TMX/SLICK-H/MCU^fl/fl^ groups subjected to HI. All mitochondria contained within each image were classified as either healthy or damaged. A total of 289 and 256 mitochondria were examined for the TMX/SLICK-H/HI and TMX/SLICK-H/MCU^fl/fl^/HI mice, respectively. Electron microscopic (EM) images in the CA1 region of the hippocampus revealed mitochondrial damage 2 h following HI for TMX/SLICK-H/HI mice that was reduced for TMX/SLICK-H/MCU^fl/fl^/HI mice (Fig. 4a–c). These morphological findings suggest that conditional MCU knockout protected mice from neuronal cell loss responsible for sensorimotor deficits after HI by reducing mitochondrial injury.

### MCU silencing reduces OGD-induced viability loss without compromising mitochondrial function

**Fig. 5:**
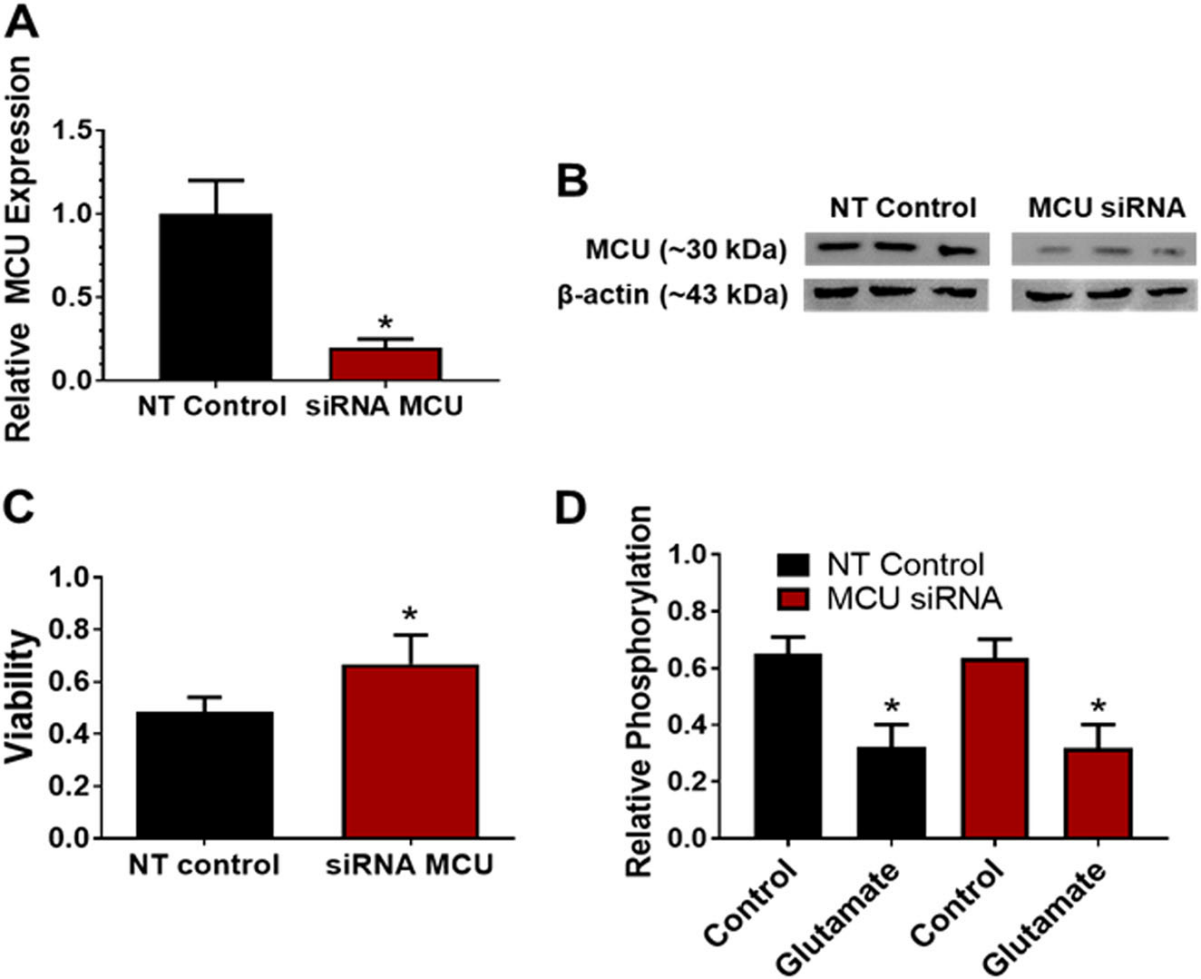
MCU knockdown protects primary cortical neuron cultures from the loss of cellular viability after OGD. **a** Relative mRNA levels for primary cortical neuron cultures 72 h after treatment with a non-targeting (NT) siRNA or siRNA against the MCU (siRNA MCU). **b** MCU protein levels for NT and siRNA MCU cortical neuron cultures. **c** Cell viability 24 h after OGD (90 min) for NT siRNA and siRNA MCU cortical neuron cultures. **d** Phosphorylation levels for pyruvate dehydrogenase (PDH) under basal and glutamate-stimulated conditions for NT siRNA and siRNA MCU cortical neuron cultures. *p < 0.05 relative to NT siRNA controls **a** and **c** or cultures not treated with glutamate (50 µM) for 30 min **d**, Mann–Whitney *U-*tests (*n* = 4)

We next examined the effects of siRNA-induced MCU silencing on the loss of cell viability in primary cortical neuron cultures 24 h following 90 min of oxygen-glucose deprivation (OGD). This experimental approach was used for two reasons: (A) Thy1 is weakly expressed during embryonic development and (B) primary cortical neuron cultures do not tolerate treatment with 4-hydroxytamoxifen^22,23^. We found that siRNA-mediated MCU knockdown reduced MCU mRNA and protein levels by 80% and 50%, respectively (Fig. 5a, b). This degree of siRNA-mediated MCU knockdown has previously been shown to decrease *N*-methyl-d-aspartate-induced mitochondrial Ca^2+^ uptake in neurons^24^. Cell viability for control cultures pretreated with the non-targeting (NT) siRNA was reduced to 48% at 24 h following OGD (Fig. 5c). Cultures pretreated with the MCU siRNA showed a significantly elevated cell viability of 71% at 24 h after OGD. These findings further support the protective effects of decreased neuronal MCU levels against ischemic/reperfusion injury.

### MCU silencing does not alter pyruvate dehydrogenase phosphorylation

Phosphorylation of the pyruvate dehydrogenase (PDH) complex is rapidly reduced by the elevation of Ca^2+^ levels in the mitochondrial matrix via the allosteric activation of Ca^2+^-sensitive PDH phosphatases^20,25,26^. We therefore measured the phosphorylation status of the PDH complex to determine whether siRNA-mediated MCU knockdown suppressed Ca^2+^-induced increases in PDH phosphatase activity. MCU silencing did not alter phosphorylation of the PDH complex (Fig. 5d). Elevation of cytosolic Ca^2+^ concentrations with glutamate (25 µM, 30 min) also produced comparable reductions in the phosphorylation of PDH for control cultures treated with either the NT siRNA or MCU siRNA (Fig. 5d). However, we cannot rule out the possibility that MCU knockdown altered the phosphorylation state of the PDH complex at earlier time points or under different Ca^2+^ loads^18,19^. Nevertheless, our findings suggest that residual MCU-mediated Ca^2+^ uptake in MCU-silenced neurons was sufficient to regulate PDH phosphorylation in the mitochondrial matrix.

### MCU knockdown does not change mitochondrial respiration or alter glycolysis

**Fig. 6:**
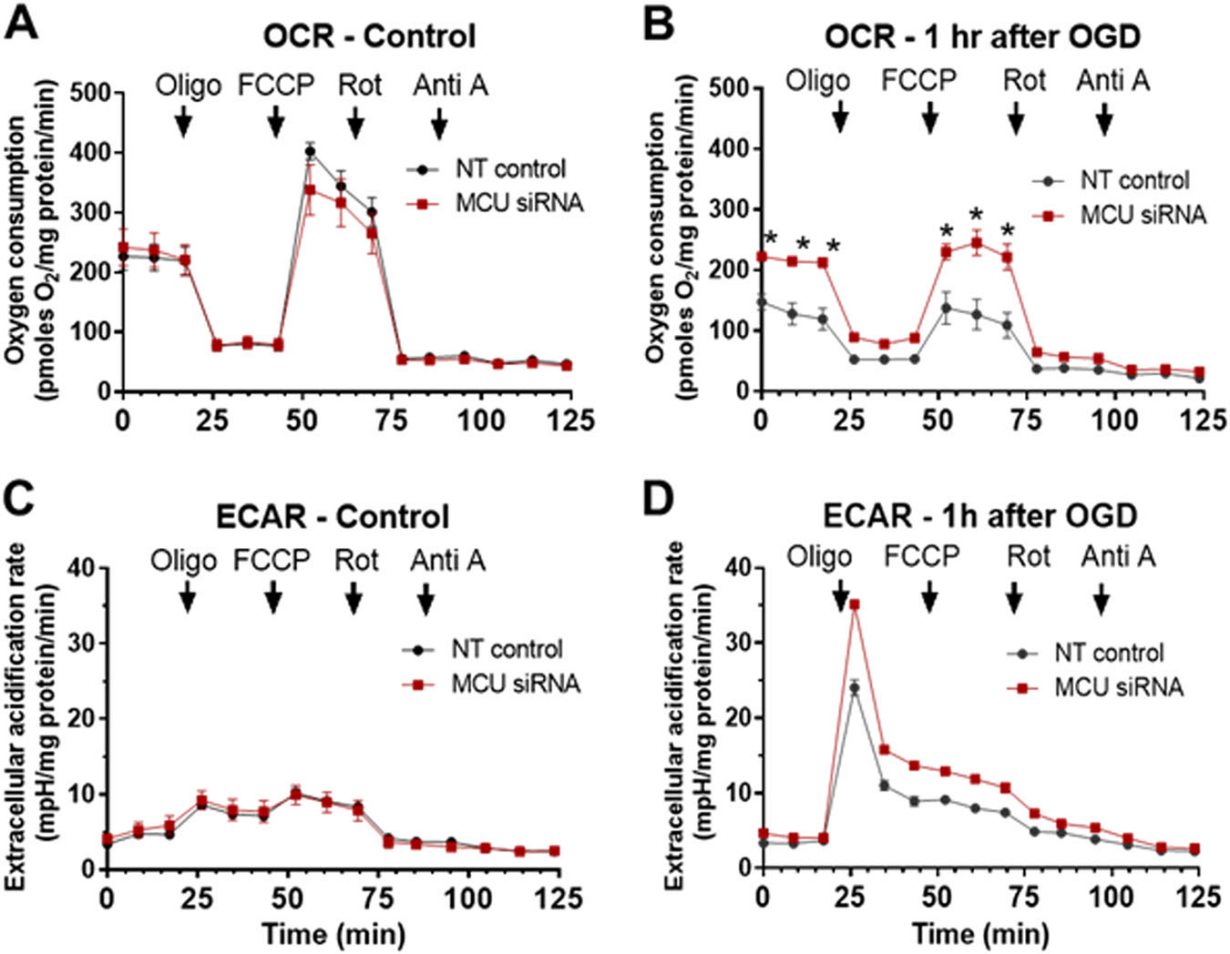
MCU silencing protects primary cortical neuron cultures from impaired mitochondrial function after OGD. Oxygen consumption rates (OCR) and extracellular acidification rates (ECAR) under control conditions **a** and **c** or 1 h after OGD **b** and **d**. Arrows indicate the addition of oligomycin (2 µM), FCCP (2 µM), rotenone (300 nM), and antimycin (**a**) (5 µM) in NT and siRNA MCU cortical neuron cultures. *p < 0.05 relative to NT siRNA controls **a** and **b**, *n* = 4. Two-way ANOVA followed by group comparisons with the Bonferroni’s post hoc test

To determine whether mitochondria function was preserved by MCU knockdown, we measured oxygen consumption rates (OCR) and extracellular acidification rates (ECAR), before and 1 h after OGD (90 min), using a Seahorse xF24 analyzer. Mitochondrial function was probed by the sequential addition of oligomycin (2 µM), FCCP (2 µM), rotenone (300 nM), and antimycin A (5 µM). OCR or ECAR levels following these treatments were comparable in control and MCU knockdown neurons before OGD (Fig. 6a, c). After OGD, both basal and FCCP-induced maximal respiration were reduced to greater extents in control than MCU-silenced neurons (Fig. 6b). ECARs following OGD were similar for the control and MCU knockdown cortical neuron cultures (Fig. 6d).

## Discussion

### Reperfusion and neuronal cell death mechanisms in HI brain injury

Adhami et al.^27^ have performed detailed studies on the mechanisms responsible for HI brain injury in adult male C57Bl/6 mice. These investigators measured cerebral flow (CBF) and arterial blood gases using a modified version of the Levine/Vannucci procedure for inducing HI brain injury. In brief, mice were anesthetized and placed on a heating pad to maintain body temperature at 36.5 to 37.5 °C. The delivery of a low oxygen (8% O_2_) gas occurred after carotid occlusion via a face mask. Using this experimental approach, Adhami et al.^27^ showed that HI caused a gradual decline of CBF to 20% of the preocclusion value. After the animals began breathing normal atmospheric air (20% O_2_), CBF returned to 40% of the preocclusion values by 30 min. Arterial oxygen saturation levels were reduced to 43% of baseline values by HI and recovered completely 30 min after the animals started breathing 20% O_2_. These findings indicate that there is partial cerebral reperfusion accompanied by complete systemic reoxygenation in the HI model. Electron microscopic, immunohistochemical and biochemical measures of the infarcted brain region have shown that HI brain injury in adult mice induces three forms of cell death—necrosis and autophagy and to a lesser extent apoptosis^27–30^. The same mechanisms of neuronal cell death have also been observed after transient forebrain ischemia produced by temporary occlusion of the middle cerebral artery^31–33^. These findings indicate that reperfusion injury occurs in the HI model resulting in the activation of similar neuronal cell death mechanisms observed following transient occlusion of the middle cerebral artery.

### Thy1-MCU deficiency confers resistance to HI-induced sensorimotor deficits and brain injury

Relative to control (TMX/SLICK-H) mice with intact MCU function, TMX-induced MCU ablation in TMX/SLICK-H/MCU^fl/fl^ mice reduced sensorimotor impairments and neuronal damage in the forebrain 24 h after HI brain injury (Fig. 1a–l). FJ staining, indicative of neuronal damage, was decreased by Thy1-MCU deficiency in the CA1 region of the dorsal hippocampus, dorsolateral striatum, and anterior motor cortex (Fig. 1c–l). Fluorescence generated by the Thy1-cre/ERT2-eYFP construct enabled us to compare the survival of eYFP^+^ neurons in TMX/SLICK-H and TMX/SLICK-H/MCU^fl/fl^ mice after HI brain injury. Quantification of eYFP^+^ cells showed that MCU deficiency in Thy1-expressing neurons also improved cell survival in the anterior motor cortex, particularly in layer V (Fig. 2a–d). At last, TTC staining revealed that Thy1-MCU deficiency reduced infarct size in the HI model of brain injury (Fig. 3a, b). TMX-induced MCU deletion in Thy1-expressing neurons at adulthood thus reduces both neuronal cell damage and loss in the forebrain that confers resistance to HI-induced sensorimotor deficits and brain injury.

### MCU ablation in Thy1-expressing neurons profoundly protects layer V pyramidal neurons

In agreement with previous findings^21–23^, the Thy1 construct produced strong eYFP^+^ labeling in layer V of the anterior motor cortex. Although less abundant, eYFP^+^ neurons were also seen in layers II–IV and VI. This localization likely accounts for the profound protection of layer V pyramidal neurons in SLICK-H/MCU^fl/fl^/HI mice. The presence of eYFP^+^ neurons in layers II-IV and VI was also consistent with decreased damage (FJ^+^ cells) in these cortical laminae for TMX/SLICK-H/MCU^fl/fl^/HI relative to TMX/SLICK-H/HI mice. The preservation of layer V pyramidal neurons was therefore a prominent neuroprotective feature of TMX/SLICK-H/MCU^fl/fl^/HI mice.

### Potential benefits of preserved layer V pyramidal neurons for sensorimotor function

Several lines of evidence suggest that the protection of layer V pyramidal neurons in TMX/SLICK-H/MCU^fl/fl^/HI mice was crucial to maintaining the sensorimotor function of these animals. Layer V pyramidal neurons play a pivotal role in the regulation movement. These neurons make distant monosynaptic contacts with neurons that comprise key motor circuits. In addition to direct corticospinal projections to the spinal cord, layer V neurons make monosynaptic contacts with neurons in the striatum, thalamus, zona incerta, and brainstem motor centers^34,35^. Collaterals from layer V pyramidal neurons also innervate the ipsilateral striatum^36,37^. Glutamatergic layer V neurons are thus strategically positioned to activate complex motor circuits that control movement. Layer V neurons display spontaneous activity that synchronizes the activity of remote motor circuits into rhythmic oscillations. For leg movements, these oscillations increase in frequency with the sequential activation of lower and upper leg muscles during walking^38^. Optogenetic studies have demonstrated that layer V pyramidal neurons initiate motor planning by synchronizing the activity of neurons in different cortical layers^39,40^. The resultant changes in rhythmic layer V firing coordinate the activation of neuromuscular circuits that power joint movements^41^. The near complete preservation of layer V neurons in TMX/SLICK-H/MCU^fl/fl^/HI mice may thus have been a major factor in preserving the sensorimotor function of these animals.

### TMX/SLICK-H/MCU^fl/fl^ mice are resistant to HI-induced mitochondrial injury

Mitochondrial Ca^2+^ overloading is an early event in the initiation of neuronal cell death in models of ischemic/reperfusion injury^42,43^. To examine the degree of mitochondrial protection achieved by Thy1-MCU deficiency, we examined the ultrastructure of mitochondria in the CA1 region of the dorsal hippocampus following HI. Relative to TMX/SLICK-H/HI mice, TMX/SLICK-H/MCU^fl/fl^/HI mice displayed a reduction in the percentage of damaged neuronal mitochondria (Fig. 4a–c). This preservation of mitochondrial ultrastructure suggests that Thy1-MCU deficiency protected neurons from HI damage by preventing mitochondrial Ca^2+^ overloading known to cause a catastrophic collapse of mitochondrial function that halts energy failure and triggers the release of multiple death-effectors^7,9^.

### MCU silencing attenuates OGD-induced viability loss without altering PDH phosphorylation

Primary cortical neuron cultures subjected to OGD were used to model ischemic/reperfusion injury in ischemic stroke^20,44,45^. The role of MCU-mediated mitochondrial Ca^2+^ overloading in ischemic/reperfusion injury was examined 24 h after a 90 min period of OGD. MCU knockdown by siRNA-induced silencing protected cortical neurons against OGD-induced viability loss (Fig. 5a–c). We have shown previously that the loss of MCU-mediated Ca^2+^ uptake in G-MCU null neurons was accompanied by elevated phosphorylation of PDH under basal and glutamate-stimulated conditions^20^. By contrast, siRNA-induced MCU silencing did not alter basal PDH phosphorylation or glutamate-induced PDH dephosphorylation (Fig. 5d). The suppression of mitochondrial Ca^2+^ uptake by MCU silencing was therefore sufficient for neuroprotection against ischemic/reperfusion injury without compromising the activity of Ca^2+^-dependent PDH phosphatases^46^. These findings indicate that siRNA-induced MCU silencing did not alter mitochondrial PDH activity.

### Knockdown of the MCU preserves mitochondrial function without enhancing glycolysis

We next compared the glycolytic rates and several aspects of mitochondrial function for respiring cortical neurons 3 days after treatment with the control (NT) siRNA or MCU siRNA. Basal respiration, ATP production, FCCP-induced maximal respiration and complex I activity were all comparable for the control and MCU siRNA-treated cortical neurons (Fig. 6a). Glycolytic rates for control and MCU siRNA-treated cortical neurons were also similar (Fig. 6c). Mitochondrial respiration and glycolysis were therefore not changed by siRNA-induced knockdown of the MCU. Next, we compared these parameters in NT siRNA- and MCU siRNA-treated cortical neuron cultures 1 h after 90 min of OGD. MCU silencing mitigated the loss of both basal and FCCP-induced maximal respiration 1 h after OGD (Fig. 6b). These results indicate the presence of a larger pool of functional mitochondria in MCU knockdown neurons after OGD. At last, glycolytic rates following OGD were similar for the NT siRNA- and MCU siRNA-treated cortical neuron cultures after OGD. MCU silencing therefore protected cortical neurons from *in vitro* ischemic/reperfusion injury with altering glycolysis.

### Neuronal MCU deficiency avoids metabolic compensations observed in G-MCU nulls

We have recently reported that G-MCU nulls are not protected from HI brain injury nor were primary cortical neuron cultures derived from these mice resistant to viability loss after OGD^20^. These findings were unexpected because Ca^2+^-induced mPTP opening was blocked in forebrain mitochondria isolated from G-MCU nulls. To resolve these findings, we demonstrated that metabolic compensations for chronically impaired mitochondrial Ca^2+^ uptake compromised the resistance of G-MCU nulls to HI brain injury^20^. Relative to WT neurons, Complex I activity was depressed in close association with elevated glycolysis in G-MCU cortical neurons by energetic stress produced by the stimulation of maximal respiratory capacity with FCCP or OGD. The depression of NADH and pyruvate levels in the hippocampi of G-MCU nulls relative to WT mice after HI further supported a metabolic switch from oxidative phosphorylation to glycolysis for energy production. In addition, PDH was hyper-phosphorylated in G-MCU null relative to WT neurons under both control and glutamate-stimulated conditions. PDH is inactivated by phosphorylation^47^. This blocks pyruvate entry into tricarboxylic acid cycle resulting in the glycolytic conversion of pyruvate to lactate in neurons by lactate dehydrogenase^48^. Based on these findings, we proposed that enhanced glycolysis after OGD deprives Complex I of reducing equivalents (NADH) required to drive oxidative phosphorylation^20^. The resultant energetic collapse could thus have promoted ischemic/reperfusion injury despite reduced mitochondrial Ca^2+^ uptake. The present findings support this mechanism by showing that acute MCU knockdown, which preserved mitochondrial function and protected neurons from ischemic/reperfusion injury, did not produce these metabolic compensations.

## Conclusions

We have shown that conditional MCU deletion in Thy1-expressing neurons protects mice from HI-induced motor deficits and forebrain damage. Relative to TMX/SLICK-H/HI mice, mitochondrial ultrastructure in the CA1 hippocampal neurons of mice was preserved in TMX/SLICK-H/MCU^fl/fl^/HI mice. This suggests that Thy1-MCU deficiency reduced HI brain damage by attenuating injurious mitochondrial Ca^2+^ overloading. Acute siRNA-mediated MCU knockdown also protected cortical neuron cultures from viability loss and mitochondrial deficits following OGD. Moreover, MCU silencing did not cause metabolic impairments previously observed in G-MCU nulls. These findings suggest that brain-penetrant MCU inhibitors are most likely to have greatest therapeutic benefit for the acute management of ischemic/reperfusion brain injury.

## Methods and materials

### Generation of central-neuron specific MCU deficient mice

Single-neuron Labeling with Inducible Cre-mediated Knockout-H (SLICK-H; The Jackson Laboratory; Stock No: 012708) mice expressing a Thy1-cre/ERT2-eYFP construct^21^ were crossed with C57Bl/6 MCU-floxed (MCU^fl/fl^) mice (generously provided by Dr. Jeffrey Molkentin, Philadelphia, Ohio, USA)^18^ to generate Thy1-cre/ERT2-eYFP^+/-^/MCU^fl/fl^ mice. These animals are hereafter referred to as SLICK-H/MCU^fl/fl^ mice. TMX (80 mg/kg) was administered once daily for 5 days by oral gavage followed by a 3-week washout period to male SLICK-H/MCU^fl/fl^ mice to produce a conditional MCU knockout in Thy1-expressing neurons, termed TMX/SLICK-H/MCU^fl/fl^ mice. SLICK-H mice which received the same TMX dosing and washout period were used as controls and termed TMX/SLICK-H mice. Age-matched male mice were used for experimentation between 8–12 weeks of age. SLICK-H mice were selected because Thy1 is expressed at adulthood in cortical and hippocampal neurons susceptible to HI brain injury. This results in strong eYFP labeling and highly-efficient Cre recombinase activity that are restricted to Thy1-expressing neurons in the central nervous system. Furthermore, SLICK-H mice do not show any gross morphological of physiological abnormalities compared with WT C57Bl/6 mice^21^.

### Western blotting

Forebrains from both TMX/SLICK-H and TMX/SLICK-H/MCU^fl/fl^ mice were homogenized in a microtube homogenizer D1030 (Sigma) and then spun at 14,000 g for 10 min (min). The supernatant was then removed followed determination of the protein concentration by the Bradford assay (Sigma). Protein samples were loaded to a sodium dodecyl sulfate polyacrylamide gel electrophoresis gel (5% stacking, 10% separating) at a concentration of 10 µg and run at 50 V for 30 min (min) followed by 100 V until the samples were approximately 1 cm from the bottom of the gel. The protein samples were then transferred onto PVDF membranes at 350 mA for 20 min. The gels were washed 3 × in TBS-T and then blocked in 5% milk for 1 h (hr). The gel was then washed 3 × with TBS-T and incubated overnight in primary antibody according to the manufacturer's instructions (anti-MCU, D2Z3B, Cell Signlaling) and (β-actin, A2066, Sigma-Aldrich). The following day the blots were washed 3 × in TBS-T and then incubated in a secondary antibody for 2 h at room temperature (anti-mouse IgG PI-2000, Vector and anti-rabbit IgG ab97051 both at 1:1000). The gels were then washed 3 × in TBS-T. An Amersham ECL prime western blotting detection kit (RPN 2232, GE healthcare) was then applied to the membrane immediately before imaging according to the manufacturer's instructions. Images were captured on a ChemiDoc Touch (BioRad). Images were then exported into Image J, where relative areas for each band were calculated. The ratio of the MCU signal to the β-actin signal was determined and these values were used to calculate a relative expression for each lane.

### HI brain damage

Mice were subjected to HI brain damage as described previously^20^. In brief, the left common carotid artery was occluded and then the mice were placed in an 8% oxygen chamber for 50 min to produce a unilateral infarct. Mice subjected to sham HI underwent anesthesia and exposure of the left carotid without occlusion. These animals were placed in the apparatus used to produce HI at normal atmospheric conditions for 50 min. Mice were examined 2 h after removal from the hypoxic chamber for EM analysis. Samples were prepared by the Electron Microscopy Core Unit at Dalhousie University as previously described^20^. Behavioral testing, Fluoro-Jade C (FJ), and TTC staining were performed 24 h following HI as previously described, in a single-blinded fashion, in accordance with the ARRIVE guidelines^20,45^. The areas occupied by eYFP^+^ neurons were also measured within a region 300 × 300 µm located in the anterior motor cortex by quantitative image analysis according to our previously described methods^20^. FJ and eYFP labeling were quantified on separate slides, as their staining protocols are not cross-compatible, in four serial sections per region for eight animals per group by an individual unaware of the treatment conditions. Two animals from each group were humanely killed prior to their desired endpoints for health reasons and thus excluded from analysis.

### Neuroscore scale: assessment of general condition and neurological deficits

A comprehensive behavioral assessment of sensorimotor deficits produced by HI brain injury was performed using a neuroscore scale developed by Dr. Ulrich Dirnagl (Charité—Universitätsmedizin Berlin, Germany). Scores ranged from 0 (healthy) to 56 (the worst performance in all categories) and represented the sum of scores for six general deficit categories (hair, ears, eyes, posture, spontaneous activity, and epileptic behavior categories) and seven focal deficits categories (body symmetry, gait, climbing on angled surface, circling behavior, front limb symmetry, compulsory circling, whisper response to light touch). A full description of these methods is available in Nichols et al.^20^.

### Assessment of mitochondrial function and glycolysis in primary cortical neuron cell cultures

Primary cortical cell cultures from C57Bl/6 mice were prepared according to our previously described methods^20^. Four days after preparing the primary neuronal cultures, either a non-targeting siRNA or siRNA against the MCU (Accell non-targeting and Accell mouse MCU (215999), GE Healthcare Bio-sciences Co) was administered at a final concentration of 1 µM for 72 h in neurobasal media. MCU RNA levels were determined by qRT-PCR according to our previously described methods^20^. Primer sequences used for qRT-PCR were as follows: β-actin, (F) - GTGACGTTGACATCCGTAAAGA, (R) - GCCGGACTCATCGTACTCC; GAPDH, (F) - AGGTCGGTGTGAACGGATTTG, (R) - GGGGTCGTTGATGGCAACA; MCU, (F) - AAAGGAGCCAAAAAGTCACG, (R) - AACGGCGTGAGTTACAAACA. The effects of OGD (90 min) on OCRs and ECARs were then examined as previously described^20^. OCRs and ECARs were normalized to protein concentrations determined using the Bio-Rad protein assay (Cat# 500-0006).

### Measurement of PDH phosphorylation and cell viability

Cortical neuron cultures were treated with vehicle (phosphate-buffered saline) or glutamate (25 µM) for 30 min, harvested and frozen until analysis (−80 °C). Phosphorylation of PDH was performed on protein extracts from the primary neuron cultures with a multiplexed ELISA kit (Cat# PDHMAG-13K, EMD Millipore) according to the manufacturer’s instructions. Primary cortical cultures were seed on 48 well-plates at a density of 150,000 neurons/well and treated with a non-targeting siRNA or MCU siRNA for 72 h followed by exposure to OGD. Cell viability was determined 24 h later using an MTT (Sigma-Aldrich, Cat#M5655-1G) assay^20^.

### Power calculations and statistical analyses

Power calculations were performed to determine the group sizes required to detect statistical differences for the animal experimentation. A group size of eight mice with a standard deviation of 45% was needed to detect an 50% difference between the means for measurements of neuroscores and neuronal cell counts an alpha level of 0.05. Mann–Whitney *U-*tests were performed to assess potential differences between neuroscores for TMX/SLICK-H and TMX/SLICK-H/MCU^fl/fl^ mice subjected to HI (Fig. 1b), Fluoro-Jade C positive cells (Fig. 1c), eYFP^+^ neurons (Fig. 2b), infarct volume (Fig. 3b), mitochondrial damage (Fig. 4c), MCU mRNA levels (Fig. 5a), cortical neuron viability loss after OGD (Fig. 5c), cortical neuron PDH phosphorylation levels (Fig. 5d). A two-way analysis of variance followed by group comparisons with the Bonferroni’s post hoc test was used to assess potential differences in OCRs and ECARs between cortical neurons treated with non-targeting siRNA or siRNA against the MCU (Fig. 6a–d).
